# Virtual touch tissue imaging and quantification (VTIQ) in the evaluation of thyroid nodules: the associated factors leading to misdiagnosis

**DOI:** 10.1038/srep41958

**Published:** 2017-02-03

**Authors:** Cheng-Yu Sun, Kai-Rong Lei, Bo-Ji Liu, Xiao-Wan Bo, Xiao-Long Li, Ya-Ping He, Dan Wang, Wei-Wei Ren, Chong-Ke Zhao, Hui-Xiong Xu

**Affiliations:** 1 Department of Medical Ultrasound, Shanghai Tenth People’s Hospital, Ultrasound Research and Education Institute, Tongji University School of Medicine, Shanghai 200072, China; 2 Department of Medical Ultrasound, Yangpu Hospital, Tongji University School of Medicine, Shanghai 200090, China; 3 Thyroid Institute, Tongji University School of Medicine, Shanghai 200072, China; 4 Shanghai Center for Thyroid Diseases, Shanghai, 200072, China

## Abstract

To evaluate the associated factors leading to misdiagnosis with VTIQ for differentiation between benign from malignant thyroid nodules (TNs). The study included 238 benign TNs and 150 malignant TNs. Conventional ultrasound (US) features and VTIQ parameters were obtained and compared with the reference standard of histopathological and/or cytological results. Binary logistic regression analysis was performed to select independent variables leading to misdiagnosis. The maximum shear wave speed (SWS) (SWS-max), mean SWS (SWS-mean), SWS-ratio and standard deviation of SWS (SWS-_SD_) were significantly higher for malignant TNs compared with benign TNs (all *P* < 0.001). SWS-mean achieved the highest diagnostic performance with a cut-off value of 3.15 m/s. False positive rate was 13.4% (32/238) while false negative rate was 35.3% (53/150). Intranodular calcification (OR: 1.715) was significantly associated with false positive VTIQ findings, while nodule size (OR: 0.936) and echotexture of the thyroid gland (OR: 0.033) were negatively associated with them. Nodule depth (OR: 0.881) and TI-RADS category (OR: 0.563) were negatively associated with false negative VTIQ findings. These US characteristic of TNs should be taken into consideration when interpreting the results of VTIQ examinations.

There is a high incidence of thyroid nodules (TNs) in the adult population^1^ and the prevalence of thyroid cancer is up to 5–15%^2^,^3^. High-frequency ultrasound (US) is the preferred non-invasive imaging modality in distinguishing malignant from benign TNs; however, there is a quite a lot overlap in imaging features between benign and malignant TNs^4^. In addition, the diagnostic performance of US depends on the operator’s experience and offers little information about mechanical properties of TNs^5^.

Shear wave (SW) imaging, also known as shear wave elastography (SWE), has been introduced into clinical practice in recent years, which provides quantitative measurement of tissue stiffness instead of qualitative stiffness information with previous strain elastography^6^,^7^. SW imaging under acoustic radiation force impulse (ARFI) excitation can be divided into two types: point shear wave speed (SWS) measurement (i.e. point SWE) and SWS imaging (i.e. two-dimensional [2D] SWE). As a point SWS measurement technique, the utility of virtual touch tissue quantification (VTQ; Siemens Medical Solutions, Mountain View, CA, USA) in distinguishing malignancy from benign TNs has been reported in numerous studies^8^,^9^,^10^,^11^. However, it does not offer a 2D stiffness map of the TNs and is not able to provide guidance for selecting SW region of interest (ROI) for quantitative measurement. Thus sometimes the SW ROI might be placed on the unwanted areas and invalid measurement results might be encountered, which is usually displayed as “X.XX m/s” and leads to confusion as to how to handle with these invalid values. In addition, the fixed SW ROI (i.e. 5 × 6 mm) is also an inherent limitation for VTQ. The surrounding thyroid parenchyma may be included in the SW ROI if the nodules are small, therefore the measurement results would be affected^8^,^12^.

The newly developed virtual touch tissue imaging and quantification (VTIQ; Siemens Medical Solutions, Mountain View, CA, USA) is a two-dimensional quantitative SWS imaging and an ARFI based method that uses an acquisition sequence consisting of reference, excitation and tracking sonic pulse types. Both reference and tracking pulses are conventional B-mode US beams that explore the difference between an initially established tissue baseline and the strain response to the radiation force generated by the excitation of a longitudinal ARFI^13^. Then the speed of SW which propagates in a perpendicular direction by a detection pulse is measured. The image is acquired by translating the pushing focus to cover up to 256 acquisition lines within 700 ms. For each, SWS is calculated at a number of locations around the pushing focus^14^. Compared with previous point SWS measurement technique, 2D SWS imaging of VTIQ might provide more precise stiffness information and avoid invalid measurements within the TNs due to its 2D visualization of SWS distribution with various colors and smaller SW ROIs (i.e. 1.5 × 1.5 mm).

Although many studies have confirmed that 2D SWS imaging improved the diagnostic performance in differentiating malignant from benign TNs^3^,^14^. Undoubtedly, some TNs might not show typical stiffness manifestations on 2D SWS imaging, which would lead to false-positive and false-negative results. To understand the factors relating to these false-positive or false-negative results is relevant in clinical practice, which may help the investigators to make correct decision when encountering a special clinical scenario. However, as far as we know, no studies have been carried out to explore the factors leading to misdiagnosis with VTIQ.

The aim of the study was to analyze the false VTIQ results in the differential diagnosis between benign and malignant TNs and to investigate the associated factors which led to false findings of TNs with VTIQ.

## Material and Methods

The Ethical Committee of Shanghai Tenth People’s Hospital approved this retrospective study and the requirement to obtain informed consent from the patients was waived due to the retrospective nature of the study. The study was performed in accordance with Declaration of Helsinki for human study.

### Patients

From June 2014 to October 2015, 385 patients with 412 TNs were retrospectively included in the study. The inclusion criteria were as follows: (1) TNs were subject to both conventional US and VTIQ examination in the referral university hospital. (2) Patients with TNs underwent fine-needle aspiration biopsy (FNAB) and/or surgery. (3) Benign TNs on FNAB without histopathological confirmation were followed up for at least 6 months. (4) Malignant TNs were confirmed by histopathological examination after surgery. (5) No invasive procedures such as thyroid surgery or FNAB were performed before. Among them, 23 patients with 24 TNs without enough thyroid tissue around the nodule at the same depth were excluded. Finally, 388 TNs in 362 patients were enrolled in the study. The patients included 99 (25.5%) men and 289 (74.5%) women. The patient mean age was 49.5 ± 12.9 years (range: 14 to 79 years). For the patient with multiple TNs, generally, the nodule which was most suspicious of malignant or the largest nodule on US was selected. For the patient with multiple suspicious nodules, all of the nodules were analyzed. Finally, single nodule in 337 patients, 2 nodules in 24 patients, and 3 nodules in 1 patient were analyzed.

### Conventional US and VTIQ examinations

Conventional US and VTIQ images were obtained using the same Siemens S3000 US scanner (Siemens Medical Solutions, Mountain View, CA, USA). The 9L4 linear array transducer (frequency range: 4–9 MHz) and/or the 14L5 linear array transducer (frequency range: 6–14 MHz) were used for conventional US examination and the 9L4 linear array transducer was used for VTIQ examination. Both of conventional US and VTIQ examinations were performed by the same operator and conventional US examination was performed before VTIQ examination. The operators were trained in terms of VTIQ examination for 2 months to ensure optimized image quality before the study.

Patient gender, age and past medical history were obtained before US examination. Patients took supine position and the image settings were adjusted to obtain optimal images. The target nodule was placed in the screen center of the US machine. On conventional US, transverse and longitudinal images were obtained for each nodule by scanning the nodule and adjacent tissue. All the US images were digitally stored and were transferred to a personal computer for further analysis.

After conventional US, SWS imaging of VTIQ was performed in the longitudinal direction of the nodule. The linear array transducer was applied very gently at the skin to avoid extra compression and held for a few seconds to stabilize the elastography images. Patients were asked to hold breath and not swallow for a few seconds while images were acquiring. The sampling box was adjusted to cover the target thyroid nodules and part of the surrounding thyroid tissue. SW quality map which reveals the signal-to-noise ratio (SNR) was acquired firstly in the target area, in which green indicates good SW quality, yellow for marginal SW quality, and red for poor SW quality^15^. Afterwards, SW velocity mode was initialed and a 2D color map of SWS distribution within the TN was obtained, in which the color represents the SWS from high (red), intermediate (yellow or green), to low (blue). SWS was measured with a small ROI box of which the diameter was 1.5 mm and the ROI size was fixed. The scale of SWS (measured in m/s) is up to 10 m/s. Cystic, necrotic and calcified areas were avoided when placing SW ROIs. ROI placement was also referenced with the SW-quality map that only the high SW quality areas were selected whereas the marginal or poor quality areas were avoided. Seven ROIs were placed on the target nodule and the ROIs were placed randomly when the velocity map showed homogeneous SW distribution; otherwise, according to the color-scale of velocity map, the ROIs were located from highest SWS to lowest SWS area (including one stiffest area and one softest area). The SWS in the surrounding thyroid tissue at the same depth were also measured for seven times. For each ROI, the machine would give the mean SWS in the ROI and displayed it on the screen. The VTIQ images were also digitally stored and were transferred to a personal computer for subsequent analysis.

### Image Interpretation and Analysis

Two other investigators retrospectively reviewed the conventional US images independently who had at least 10 years of experience in thyroid US and 2 years of experience in thyroid elastography. Different opinions were solved with consensus. The TNs were evaluated for the following US features: capsule contact (no contact, former capsule contact, rear capsule contact, beside trachea, beside carotid), ratio of perimeter in contact with the capsule (no contact, less than 25% of perimeter, 25%–50% of perimeter, more than 50% of perimeter in contact with the capsule), size (the largest diameter), depth (distance from the skin to the center of the TN), halo sign (present or not), margin (circumscribed, not circumscribed), echogenicity (mixed-, hyper-, iso-, hypo, and marked hypo-echogenicity; hypoechogenicity means the echogenicity is lower than the surrounding thyroid tissue, while marked hypoechogenicity means the echogenicity is lower than the nearby strap muscle), internal components (completely solid or complex cystic and solid including three categories: cystic portion up to 25%, cystic portion of 25%–50%, cystic portion of more than 50%), posterior acoustic feature (none, enhancement, shadowing), intranodular calcification (non-calcification; microcalcification, less than or equal to 1 mm in diameter; macrocalcification, greater than 1 mm in diameter; when TN had both microcalcification and macrocalcification, it was defined as microcalcification), intranodular vascularity (type I, absence of blood flow; type II, slight internal flow without peripheral flow; type III, slight peripheral flow without internal flow; type IV, marked peripheral flow with slight internal flow; type V, marked internal flow with slight peripheral flow), location of TNs (left lobe, right lobe, isthmus), position of TNs (the upper, the middle part, the lower part of the thyroid gland), shape (wider than tall, taller than wide), thyroid enlargement (present or not), single nodule or multiple nodules. The nodules were classified according to thyroid imaging reporting and data (TI-RADS) system described by Kwak *et al*.^16^ and the suspicious US features of solid component, hypoechogenicity or marked hypoechogenicity, irregular or microlobulated margin, taller than-wide shape and microcalcifications were applied to categorize TNs: TI-RADS category 3 (no suspicious US features), 4a (one suspicious US feature), 4b (two suspicious US features), 4c (three or four suspicious US features), and 5 (five suspicious US features). The echotexture of the thyroid background was categorized as homogeneous or heterogeneous.

Four values of SWS were computed for analysis: the mean SWS (SWS -mean) for the seven ROIs in TN, the maximum SWS (SWS-max) for the seven ROIs, standard deviation of SWS (SWS-_SD_) for the seven ROIs, and the SWS ratio (SWS-ratio) which is the ratio of SWS-mean value of TNs to the SWS-mean of the surrounding thyroid tissue.

### Reference Standard

For malignant TNs, only the histopathological results after surgery was used as the reference standard; while for benign TNs, besides histopathological results after surgery, Bethesda category 2 cytological results on FNAB and followup for at least 6 months without change in size and US appearance was used as the reference standard.

### Statistical analysis

Statistical analysis was performed with the SPSS 20.0 software (SPSS, Chicago, IL, USA) and MedCalc software (Mariakerke, Belgium). Means and SD were used for continuous data. Comparison of the values of SWS (including SWS-max, SWS-mean, SWS-_SD_ and SWS-ratio) in benign and malignant TNs were conducted using independent *t* test. Receiver operating characteristic (ROC) curve was applied to obtain the optimal SWS cut-off value and the area under ROC curve (Az). Binary logistic regression analysis was performed to evaluate independent characteristics related to false VTIQ findings. Univariate analysis was performed first, and then the statistically significant variables (P < 0.05) were put into the model of binary logistic regression analysis with a forward stepwise selection method. *P* values of less than 0.05 were considered statistically significant and the independent variables with *P* values less than 0.05 were selected for partial regression coefficient (β), odds ratio (OR) estimates and 95% confidence interval (CI).

## Results

There were 150 (38.7%) malignant TNs and 238 (61.3%) benign TNs. All the 150 malignant TNs were confirmed by histopathological examinations after surgery. The pathological results of the malignant TNs were as follows: papillary thyroid carcinoma (PTC) (n = 144), medullary carcinoma (n = 3), follicular carcinoma (n = 2), and nondifferentiated carcinoma (n = 1). For the 238 benign TNs, 70 were confirmed by histopathological examinations after surgery, while the remaining 168 TNs were confirmed by Bethesda category 2 cytological results on FNAB and followup for at least 6 months without change in size and US appearance.

All the quantitative VTIQ parameters (including SWS-mean, SWS-max, SWS-_SD_ and SWS-ratio) were significantly higher for the malignant TNs compared with those for the benign TNs (all *P* < 0.001) (Table 1). Among the VTIQ parameters, SWS-mean with a cut-off value of 3.15 m/s achieved the highest Az value, with a sensitivity of 64.7% (97/150), specificity of 86.6% (206/238), accuracy of 78.1% (303/388), PPV of 75.2% (97/129), and NPV of 79.5% (206/259) (Table 2). Benign TNs that had an SWS-mean ≤ 3.15 m/s were classified as “true negative” whereas those with SWS-mean >3.15 m/s were classified as “false positive”. Conversely, malignant TNs that had an SWS-mean ≤ 3.15 m/s were classified as “false negative” whereas those with SWS-mean >3.15 m/s were classified as “true positive”. The false positive rate was 13.4% (32/238) and the false negative rate was 35.3% (53/150).

The results of univariate logistic regression analysis are summarized in Table 3. For benign TNs, the significant features for differentiation between false positive VTIQ and true negative VTIQ results were as follows: echotexture of the thyroid background, position of capsule contact (especially beside carotid), ratio of perimeter in contact with the capsule, size, halo sign, margin, echogenicity, internal components, posterior acoustic feature, intranodular calcification, intranodular vascularity (all *P* < 0.05). For malignant TNs, the significant features for differentiation between false negative VTIQ and true positive VTIQ results were as follows: age, lesion depth, position of capsule contact, ratio of perimeter in contact with the capsule, echogenicity, posterior acoustic feature, intranodular calcification, intranodular vascularity of TNs, TI-RADS category (all *P* < 0.05).

**Figure 1:**
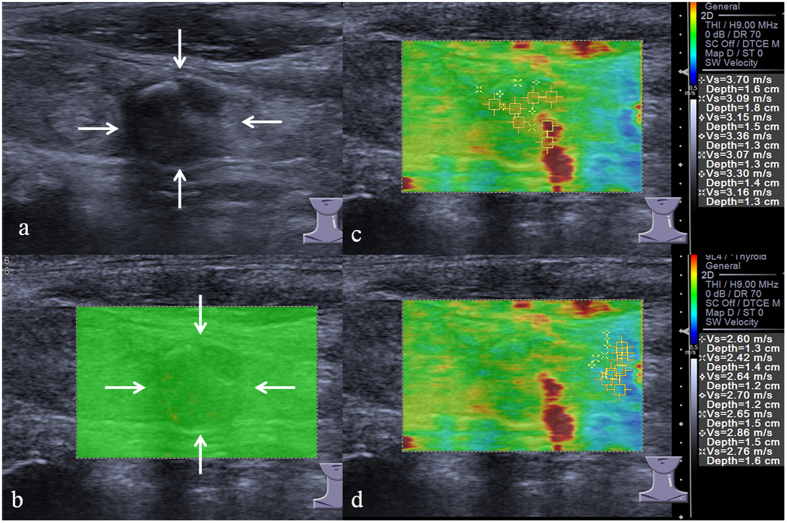
VTIQ Images acquired in a 62-year-old man. Fine-needle aspiration biopsy for this TN is benign. (**a**) Conventional ultrasound shows a 10.8-mm TN that appears hypoechogenicity, macrocalcification, regular shape and wider than tall. (**b**) SW-quality image: green color represents a good image quality. (**c**) The SWS measurement of TN. SWS-mean is 3.26 m/s and SWS-_SD_ is 0.22. (**d**) The SWS measurement of the adjacent thyroid parenchyma. SWS-mean is 2.66 m/s. VTIQ = Virtual Touch Imaging Quantification; TN = thyroid nodule; SW = shear wave; SWS = shear wave speed; SD = standard deviation.

**Figure 2:**
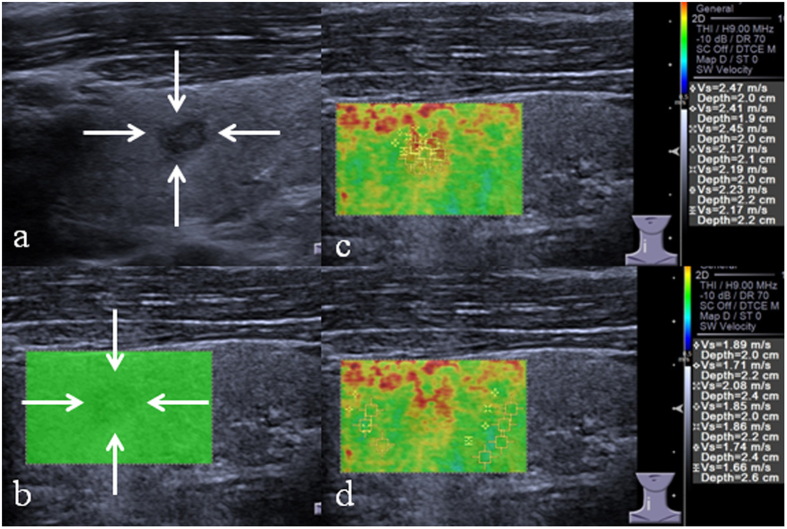
VTIQ Images acquired in a 27-year-old man. Surgical pathology confirms the diagnosis of papillary carcinoma. (**a**) Conventional ultrasound shows a 5.3-mm TN classified as TI-RADS 4a on B-mode ultrasound that appears hypoechogenicity, lacking microcalcification, regular shape and wider than tall. (**b**) SW-quality image: green color represents a good image quality. (**c**) The SWS measurement of TN. SWS-mean is 2.29 m/s SWS-_SD_ is 0.14. (**d**) The SWS measurement of the adjacent thyroid parenchyma. SWS-mean is 1.83 m/s. VTIQ = Virtual Touch Imaging Quantification; TN = thyroid nodule; SW = shear wave; SWS = shear wave speed; ROI = region of interest; SD = standard deviation.

Binary logistic regression analysis showed that intranodular calcification (β: 0.539, OR: 1.715, 95% CI: 1.046–2.811) were independent characteristics related to false positive VTIQ findings, while nodule size (β: −0.067, OR: 0.936, 95% CI: 0.881–0.994) and echotexture of the thyroid background (β: −3.410, OR: 0.033, 95% CI: 1.217–5.617) were negatively associated with false positive findings (Fig. 1) (Table 4). On the other hand, nodule depth (β: −0.126, OR: 0.881, 95% CI: 0.800–0.970) and TI-RADS category (β: −0.575, OR: 0.563, 95% CI: 0.339–0.933) were negatively associated with false negative VTIQ findings (Table 5) (Fig. 2). False negative VTIQ findings were as follows: PTC (n = 49; 34.0%, 49/144), medullary carcinoma (n = 2; 66.7%, 2/3), follicular carcinoma (n = 2; 100%, 2/2), nondifferentiated thyroid carcinoma (n = 0; 0%, 0/1).

## Discussion

Tissue stiffness is one of the characteristics that may reflect the nature of TNs^17^. US elastography as a diagnostic tool can assess the tissue stiffness, which helps differentiation between benign and malignant TNs^8^,^18^. We investigated the VTIQ parameters including SWS-mean, SWS-max, SWS-_SD_ of TNs and SWS-ratio and all of them were significantly higher for malignant TNs compared with benign ones. SWS-mean with cut-off value of 3.15 m/s had the highest Az value and achieved 64.7% sensitivity, 86.6% specificity, 75.2% PPV, 79.5% NPV, and 78.1% accuracy. Ghobad *et al*.^19^ reported 79.27% sensitivity, 71.52% specificity, 26.75% PPV and 96.34% NPV for predicting thyroid cancer with a cut-off SWS-max value of 3.54 m/s. In the current study, the cut-off SWS-max value was 3.61 m/s, which was consistent with Ghobad’s result.

In this series, The false positive rate was 13.4% and the false negative rate was 35.3%. Xu *et al*.^20^ reported that, with a threshold mean SWS of 2.87 m/s applying VTQ, the false negative rate was 31.8% (21 of 66), and the false positive rate was 23.1% (27 of 117). The false positive rates in this series were lower than those in their studies, which may be ascribed to the fact that quality measure and smaller SW ROI were applied in the current study. Despite the relatively high false results, the specificity of VTIQ was 86.6% with SWS-mean and 93.7% with SWS-max, which was in agreement with a recent consensus of World Federation for Ultrasound in Medicine and Biology (WFUMB) panel group that SWS imaging may be useful in selecting patients with TNs for surgery and SWS imaging can be considered as a useful complement to conventional ultrasound^21^. Compared with conventional US, SWS imaging has improved specificity for diagnosing malignant TNs and also improved the confidence for diagnosis^21^.

Binary logistic regression analysis showed that intranodular calcification (OR: 1.715) was independent characteristic associated with false positive findings on VTIQ. Out of 144 calcified benign TNs, 17.4% had an SWS-mean >3.15 m/s while only 7.4% of the 94 non-calcified TNs. Veyrieres^22^ reported that 25.6% calcified TNs were wrongly classified by elastography while 9.13% for non-calcified TNs. The false positive cases for calcified benign TNs is lower in our study, which might be due to the smaller SW ROIs for VTIQ. The small ROI can minimize the influence of calcifications to SWS measurement, however, the influence can not be avoided when diffuse calcifications are present. Calcifications would increase the risk for malignancy^23^, therefore, for TNs with calcification, image analysis with combination of US elastography and conventional US is necessary.

On the other hand, nodule size (OR: 0.936) was negatively associated with false positive findings on VTIQ, which means false positive results were apt to be encountered in smaller TNs. Fukuhara *et al*.^24^ found the dispersion of VTQ of benign TNs and PTCs tended to be greater for small nodules and concluded that SWE is not suitable for measurement of small nodules. The underlying mechanism might be that the ultrasonic waves of the push pulse were reflected and refracted at the curved boundaries of the nodules, resulting in irregular production of shear waves.

It is interesting to find that echotexture of the thyroid gland (OR: 0.033) was negatively associated with false positive findings on VTIQ, which indicates that benign TNs with heterogeneous thyroid gland was not easy to be misdiagnosed. Han *et al*.^25^ reported that the stiffness of extra-nodular thyroid tissue instead of TNs was affected by the severity of chronic autoimmune thyroiditis, but there is no significant difference of SWS of benign and malignant TNs in the comparison between the chronic autoimmune Hashimoto’s thyroiditis (HT) group and the non-HT group. Therefore, the heterogeneous thyroid background does not increase of the stiffness of benign TNs whereas faciliates correct diagnosis for those TNs.

Nodule depth (OR: 0.881) and TI-RADS category (OR: 0.563) were identified to be negatively associated with false negative results with VTIQ. In other words, false negative results were inclined to be found in superficial TNs and those with lower TI-RADS category. Hong *et al*. reported that malignant TNs protruded from the anterior capsule of thyroid might be elastic because the reference surrounding tissues were strap muscles instead of the thyroid parenchyma^26^. However, for deeper TNs, the stress transmission is attenuated with increasing distance from the transducer, which might result in artefactual tissue “hardening”^27^. In addition, malignant tumors are usually more heterogeneous than benign ones^28^,^29^,^30^. In the current study, the average SWS-_SD_ values of TNs which were assigned to TI-RADS categories 3, 4a, 4b, 4c were 0.11, 0.30, 0.49 and 0.67, respectively. The higher TI-RADS categories, the more obvious the heterogeneity of TNs is. This may be the reason why false negative malignant TNs are inclined to be lower TI-RADS categories since TNs with lower TI-RADS categories are usually be homogenous.

Different pathological types of malignant TNs might have different hardness. PTC, which is the most common thyroid malignancy, tends to be hard. PTC has complex papillae with a central fibrovascular stalk. And psammoma bodies and fibrosis are often found in them^26^. However, 34% (49/144) PTCs in our series showed lower SWS values, which might be associated with the high percentage of microcarcinomas (100 of 150, 66.7%) and microcarcinomas usually have homogeneous pathological components^31^. Two follicular carcinomas (100%) in the current study were soft. In a meta-analysis by Sun *et al*.^32^, a total of 7 studies reported 11 follicular carcinomas, and 10 were erroneously diagnosed as benign nodules with elastography. The reason may also be that follicular carcinoma has homogeneous pathological components^32^. Medullary and nondifferentiated carcinoma may also be varied in stiffness^33^. In our study, 66.7% (2/3) medullary carcinomas showed lower SWS values on VTIQ. However, the case numbers with follicular carcinoma, medullary carcinoma and nondifferentiated thyroid carcinoma were small, thus future study is necessary to evaluate the relationship between pathological types of malignant TNs and tissue stiffness.

This study had several limitations, Firstly, retrospective nature of the study would decrease the power of the study thus future prospective study is mandatory to verify the results. Secondly, selection bias may exist because the study included only patients with symptoms or suspicious US characteristics of malignancy who were scheduled for FNAB or surgery, accordingly, the rate of malignancy (150 of 388 nodules) is higher than that of general population^19^. Thirdly, the sample size of misdiagnosed TNs is small on VTIQ. In addition, some benign nodules were confirmed by cytological results from FNAB and followup. Some of these TNs may be malignant, although it is reported that the risk of malignant TNs following a benign biopsy is less than 3%^34^. However, it is not ethical to perform surgery to all the benign nodules thus this limitation is hardly avoided.

In conclusion, false positive and false negative results may be encountered in some TNs when VTIQ alone is applied. US features such as intranodular calcification, small nodule and homogeneous thyroid backgroud is associated with the false positive VTIQ findings. Superficial TNs and those with lower TI-RADS category are likely to show false negative VTIQ findings. These US characteristic of TNs should be taken into consideration when interpreting the results of VTIQ examinations.

## Additional Information

**How to cite this article**: Sun, C.-Y. *et al*. Virtual touch tissue imaging and quantification (VTIQ) in the evaluation of thyroid nodules: the associated factors leading to misdiagnosis. *Sci. Rep.* **7**, 41958; doi: 10.1038/srep41958 (2017).

**Publisher's note:** Springer Nature remains neutral with regard to jurisdictional claims in published maps and institutional affiliations.

## Figures and Tables

**Table 1:** SWS measurement results on VTIQ in benign and malignant thyroid nodules.

Thyroid Nodule	n	SWS-mean (m/s)	SWS-max (m/s)	SWS- _SD_ (m/s)	SWS-ratio
Benign	238	2.63 ± 0.44 (1.53–4.03)	2.95 ± 0.48 (1.75–4.53)	0.23 ± 0.12 (0.03–0.70)	1.08 ± 0.14 (0.58–1.58)
Malignant	150	3.71 ± 1.24 (1.87–8.74)	4.27 ± 1.62 (2.07–9.75)	0.43 ± 0.37 (0.03–2.12)	1.32 ± 0.36 (0.91–3.49)
P Value		<0.001*	<0.001*	<0.001*	<0.001*

Data are means ± standard deviations, and the value ranges are in parentheses. SWS = shear wave speed, VTIQ = virtual touch tissue imaging and quantification. *Indicates statistically significant difference.

**Table 2:** Diagnostic performance of SWS imaging parameters on VTIQ.

Parameters	Cut-off value	Sensitivity (%)	Specificity (%)	Accuracy (%)	PPV (%)	NPV (%)	Az (95% CI)	*P* value
SWS-mean	3.15 m/s	64.7 (97/150)	86.6 (206/238)	78.1 (303/388)	75.2 (97/129)	79.5 (206/259)	0.827 (0.785–0.863)	<0.001*
SWS-max	3.61 m/s	60.0 (90/150)	93.7 (223/238)	80.7 (313/388)	85.7 (90/105)	78.8 (223/283)	0.814 (0.722–0.852)	<0.001*
SWS-_SD_	0.34 m/s	47.3 (71/150)	85.3 (203/238)	70.6 (274/388)	67.0 (71/106)	72.0 (203/282)	0.682 (0.633–0.728)	<0.001*
SWS-ratio	1.12	76.7 (115/150)	65.1 (155/238)	69.6 (270/388)	58.1 (115/198)	81.6 (155/190)	0.774 (0.729–0.815)	<0.001*

SWS = shear-wave speed; VTIQ = virtual touch tissue imaging and quantification; PPV = positive predictive value; NPV = negative predictive value. *Indicates statistically significant difference.

**Table 3:** Analyzing the correlation of patient and morphologic characteristics of the TNs with SWS imaging findings by univariate logistic regression analysis.

Characteristics	Benign TN			Malignant TN		
	SWS-mean			SWS-mean		
	<3.15 m/s (n = 206)	≥3.15 m/s (n = 32)	*P* value	<3.15 m/s (n = 53)	≥3.15 m/s (n = 97)	*P* value
Age (years)*	51.4 ± 12.0	51.0 ± 11.0	0.065	46.0 ± 14.0	46.9 ± 14.1	0.046
Depth (mm) *	16.0 ± 4.6	14.0 ± 2.8	0.993	17.0 ± 3.1	14.0 ± 3.8	0.032
Thyroid background			<0.001			0.290
Homogeneous	153 (74.3)	17 (53.1)		42 (79.2)	59 (60.8)	
Heterogeneous	53(25.7)	15(46.9)		11 (20.8)	38 (39.2)	
Position contacted capsule			0.001			0.009
None	88 (42.7)	10 (31.2)		35 (66.0)	37 (38.1)	
Former capsule	47 (22.8)	9 (28.1)	0.291	8 (15.1)	19 (19.6)	
Rear capsule	59 (28.6)	9 (28.1)	0.383	5 (9.4)	22 (22.7)	
Beside trachea	10 (4.9)	2 (6.3)	0.908	3 (5.7)	14 (14.4)	
Beside carotid	2 (1.0)	2 (6.3)	0.039	2 (3.8)	5 (5.2)	
Contact with the capsule			<0.001			0.022
No	88(42.7)	10(31.3)		23(43.4)	47(48.5)	
<25%	44(21.4)	12(37.5)		16(30.2)	32(33.0)	
≥25%, <50%	65(31.6)	9(28.1)		13(24.5)	16(16.5)	
≥50%	9(4.4)	1(3.1)		1(1.9)	2(2.1)	
Size (mm)*	15.0 ± 11.0	10.3 ± 5.7	0.005	9.8 ± 5.7	10.5 ± 7.9	0.129
Halo sign			0.043			0.172
Absent	147 (71.4)	27 (84.4)		47 (88.7)	94 (96.9)	
Present	59 (28.6)	5 (15.6)		6 (11.3)	3 (3.1)	
Margin			0.006			0.167
Circumscribed	167 (81.1)	27 (84.4)		16 (30.2)	20 (20.6)	
Not circumscribed	39 (18.9)	5 (15.6)		37 (69.8)	77 (79.4)	
Echogenicity			0.001			0.042
Marked hypoechoic	30(14.6)	9(28.1)		32(60.4)	60(61.9)	
Hypoechoic	109(52.9)	15(46.9)		17(32.1)	29(29.9)	
Isoechoic	28(13.6)	3(9.4)		1(1.9)	4(4.1)	
Hyperechoic	2(1.0)	0(0.0)		0	0(0.0)	
Mixed echoic	37(18.0)	5(15.6)		3(5.7)	4(4.1)	
Internal components			<0.001			0.257
Solid	169(82.0)	27(84.4)		50(94.3)	93(95.9)	
Cystic portion 0–25%	22(10.7)	3(9.4)		3(5.7)	4(4.1)	
Cystic portion 25–50%	7(3.4)	2(6.3)		0(0.0)	0(0.0)	
Cystic portion ≥ 50%	8(3.9)	0(0.0)		0(0.0)	0(0.0)	
Posterior acoustic feature			<0.001			0.019
None	122(59.2)	20(62.5)		33(62.3)	68(70.1)	
EnhancementEnhancement	46(22.3)	4(12.5)		5(9.4)	6(6.2)	
Shadowing	38(18.4)	8(25.0)		15(28.3)	23(23.7)	
Calcification			<0.001			0.046
No	87(42.2)	7(21.9)		14(26.4)	27(27.8)	
Microcalcification:	72(35.0)	14(43.8)		27(50.9)	55(56.7)	
Macrocalcification	47(22.8)	11(34.4)		12(22.6)	15(15.5)	
Intranodular vascularity			0.014			0.040
Type Ι	36(17.5)	7(21.9)		18(34.0)	24(24.7)	
Type II	42(20.4)	10(31.3)		12(22.6)	28(28.9)	
Type ΙII	34(16.5)	7(21.9)		11(20.8)	12(12.4)	
Type ΙV	64(31.1)	6(18.8)		4(7.5)	14(14.4)	
Type V	30(14.6)	2(6.3)		8(15.1)	19(19.6)	
TI-RADS category			0.096			0.041
3	67(32.5)	6(18.8)		1(1.9)	1(1.0)	
4a	137(66.5)	25(78.1)		30(56.6)	35(36.1)	
4b	1(0.5)	1(3.1)		16(30.2)	47(48.5)	
4c	1(0.5)	0(0.0)		6(11.3)	14(14.4)	

TN = thyroid nodule; SWS = shear-wave speed; TI-RADS = thyroid imaging reporting and data system. *Data are means ± standard deviations.

**Table 4:** Evaluating independent factors related to false VTIQ finding of benign TNs by binary logistic regression analysis.

Characteristics	β	OR	95% CI	P value
Thyroid background	−3.410	0.033	1.217, 5.617	<0.001*
Size	−0.067	0.936	0.881, 0.994	0.031*
Calcification	0.539	1.715	1.046, 2.811	0.032*
Position contacted capsule	−0.097	0.908	0.628, 1.312	0.606
Contact with the capsule	0.298	1.347	0.695, 2.608	0.377
Halo sign	−0.375	0.687	0.283, 1.668	0.407
Echogenicity	0.414	1.513	0.774, 2.961	0.226

VTIQ = virtual touch tissue imaging and quantification; TN = thyroid nodule; OR = odd ratio; CI = confidence interval. *Indicates statistically significant difference.

**Table 5:** Evaluating the independent factors related to false VTIQ finding of malignant TNs binary logistic regression analysis.

Characteristic	β	OR	95%CI	P value
Depth	−0.126	0.881	0.800, 0.970	0.010*
TI-RADS category	−0.575	0.563	0.339, 0.933	0.026*
Age	2.067	7.898	0.319, 0.974	0.139
Contact with the capsule	−0.056	0.946	0.669, 1.337	0.751

VTIQ = virtual touch tissue imaging and quantification; TN = thyroid nodule; OR = odd ratio; CI = confidence interval; TI-RADS = thyroid imaging reporting and data system. *Indicates statistically significant difference.
